# Generalized Vitiligo Following Radiation Therapy for a B2 Thymoma: A Case Report

**DOI:** 10.7759/cureus.37538

**Published:** 2023-04-13

**Authors:** Jordin Sirody, Diane Orlinsky, Eva Simmons-O’Brien

**Affiliations:** 1 Medicine, University of Maryland School of Medicine, Baltimore, USA; 2 Dermatology, Simmons-O’Brien & Orlinsky, Towson, USA

**Keywords:** vitiligo, general dermatology, clinical dermatology, radiation oncology education, malignant thymoma, segmental vitiligo

## Abstract

Vitiligo is an idiopathic skin disorder of multifactorial etiology that is characterized by skin depigmentation. Generalized vitiligo following radiation therapy has rarely been reported in the literature. The mechanism underlying radiation-induced disseminated vitiligo is not yet fully understood. However, multiple factors, including genetic susceptibility and autoimmunity, are likely involved in the pathogenesis of the condition. We report a case of disseminated vitiligo in a patient with no preexisting personal or family history of the condition following three months of localized radiation therapy to the mediastinum.

## Introduction

Vitiligo is an idiopathic skin condition characterized by depigmented macules and patches that occurs in about 0.5%-1% of the world’s population [1]. The condition affects all genders, ages, races, and ethnicities. Multiple factors, including genetics and autoimmunity, are likely involved in the development of vitiligo [2]. While the exact mechanism of depigmentation remains unclear, its pathogenesis involves the destruction of epidermal melanocytes, with recent consensus suggesting an autoimmune nature [3]. We report a rare case of generalized depigmentation in a patient with no prior history of vitiligo after receiving radiation therapy for a thymoma. To date, few published cases of novel depigmentation following radiation in patients with no history of vitiligo exist in the literature. Of these cases, only two report dissemination of the depigmentation beyond the site of irradiation [4]. This unusual occurrence has significance in gaining a better understanding of the potential adverse cosmetic and psychological consequences of radiation therapy.

## Case presentation

**Figure 1:**
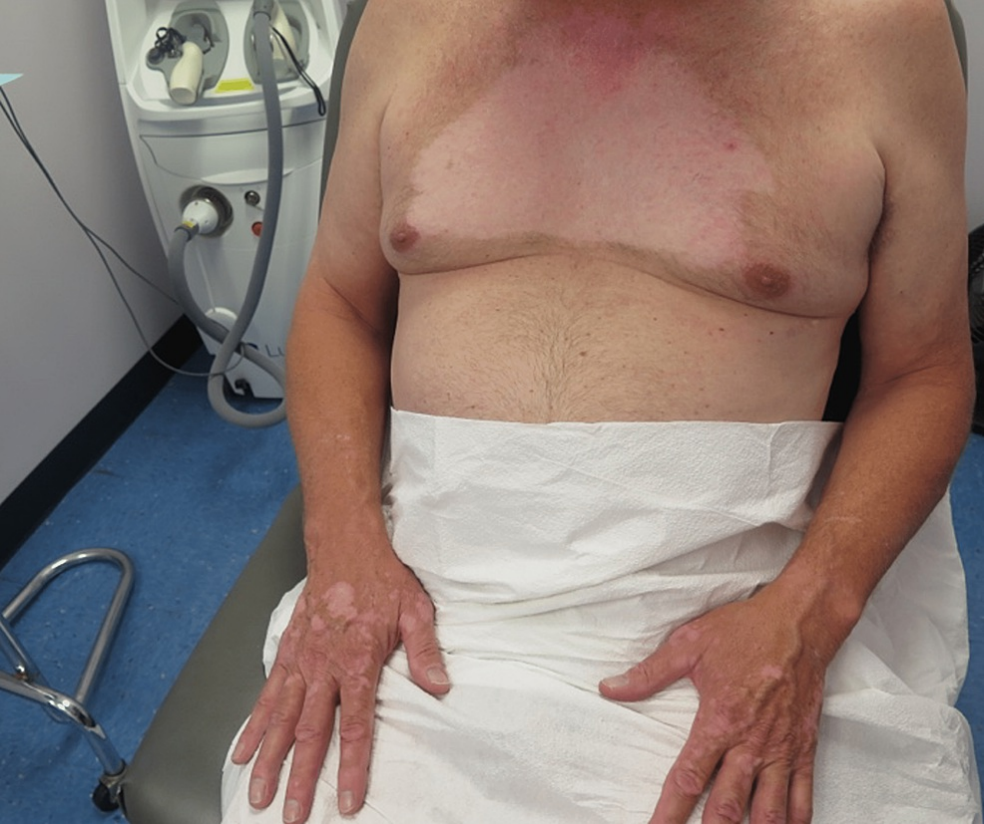
Depigmentation of the chest

**Figure 2:**
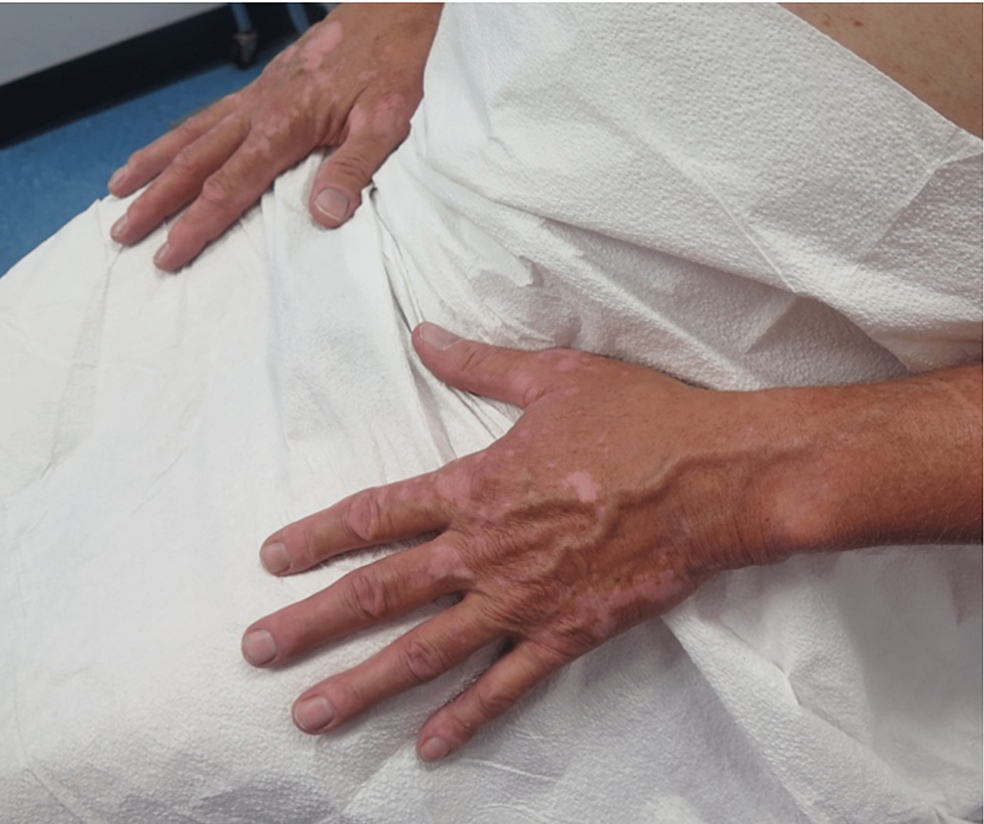
Depigmentation of both hands

A 57-year-old healthy Caucasian man with no significant past medical history presented to his primary care physician for an annual physical exam. The patient was asymptomatic but had a palpable mass on his neck that was detected by his physician. After visualization of a large mediastinal mass on CT imaging, the patient underwent a thymectomy in January 2018. Surgical pathology revealed a type B2 thymoma. He then received three months of localized radiation therapy delivered in eight doses to the mediastinum for a total cum dose of 5400 cGy in 30 fractions. No adjuvant chemotherapy was given. Toward the end of his radiation treatment, the patient reported local depigmentation on his chest (Figure 1). Within three months, the depigmentation spread to his hands, which had not been exposed to radiation (Figure 2). Subsequently, the depigmentation remained stable for three years. The patient had no skin rashes, lesions, or inflammation before the onset of the pigment changes. He denied any history of viral or bacterial illness during his radiation treatment or the immediate months that followed. The patient has no personal or family history of vitiligo or any other skin disorders. Wood's lamp examination of the lesions revealed depigmentation. A clinical diagnosis of vitiligo was made. The patient did not elect to pursue any treatment for his condition. 

## Discussion

The mechanism of radiotherapy-induced disseminated vitiligo is not yet understood. Prior studies have described Koebner’s phenomenon, in which skin injury such as irradiation prompts new depigmented lesions in patients with a prior history of vitiligo [5]. However, our patient had no prior history. A “two-hit” hypothesis has been proposed for the development of vitiligo [6]. Localized radiotherapy creates oxidative stress that results in the formation of neoantigens that then elicit an autoimmune response to melanocytes in genetically susceptible hosts, causing destruction and depigmentation [6]. Other mechanisms of physical destruction have also been proposed, including apoptosis generated by free radicals and secondary messengers like ceramide [7]. The concordance rate of vitiligo in monozygotic twins is 23%, supporting the genetic nature of vitiligo while also highlighting the importance of additional environmental insults in its pathogenesis [8]. It is, therefore, possible that the development of generalized vitiligo requires a combination of genetic susceptibility and an autoimmune inciting event.

The onset of vitiligo has also been reported following major infections, as well as other stimuli such as pregnancy, trauma, and stress, all of which have been theorized to result in the overproduction of reactive oxygen species [9]. UV radiation has also been shown to be an environmental cause of excess reactive oxygen species production [10]. Melanocytes are particularly vulnerable to oxidative stress relative to other cell types due to reduced catalase and glutathione peroxidase activity [11]. Considering the timeline of our patient’s vitiligo and the location of origin, irradiation is the most likely inciting event. However, it is also possible that another factor, such as an undetected infection or stress, triggered its onset.

## Conclusions

Although disseminated vitiligo is an exceptionally rare occurrence following irradiation, patients should be counseled on the possibility of its development as part of a full informed consent process. Vitiligo is a difficult-to-treat condition that is often cosmetically distressing to patients. Further studies are needed to identify patients who may be more likely to develop vitiligo following physical trauma, such as irradiation. Future investigations should explore whether patients with radiotherapy-induced vitiligo or other inciting physical traumas are also at elevated risk for the development of other autoimmune disorders.
